# The Effect of Buckwheat Resistant Starch on Intestinal Physiological Function

**DOI:** 10.3390/foods12102069

**Published:** 2023-05-21

**Authors:** Zhan-Bin Sun, Xiao Zhang, Yi Yan, Jia-Liang Xu, Xin Lu, Qing Ren

**Affiliations:** 1 School of Light Industry, Beijing Technology and Business University, Beijing 100048, China; 2 Key Laboratory of Brewing Molecular Engineering of China Light Industry, Beijing 100048, China; 3 State Key Laboratory for Infectious Disease Prevention and Control, National Institute for Communicable Disease Control and Prevention, Chinese Center for Disease Control and Prevention, Beijing 102206, China

**Keywords:** buckwheat-resistant starch, intestinal health, physicochemical functionality, intestinal microbe

## Abstract

Resistant starch appears to have promising effects on hypertension, cardiovascular and enteric illness. The influence of resistant starch on intestinal physiological function has drawn great attention. In this study, we first analyzed the physicochemical characteristics, including the crystalline properties, amylose content, and anti-digestibility among different types of buckwheat-resistant starch. The influence of resistant starch on the physiological functions of the mouse intestinal system, contained defecation, and intestinal microbes were also evaluated. The results showed that the crystalline mold of buckwheat-resistant starch changed from A to B + V after acid hydrolysis treatment (AHT) and autoclaving enzymatic debranching treatment (AEDT). The amylose content in AEDT was higher than in AHT and raw buckwheat. Moreover, the anti-digestibility of AEDT was also stronger than that in AHT and raw buckwheat. The buckwheat-resistant starch can promote bowel intestinal tract movement. The quantity of intestinal microbe was regulated by buckwheat-resistant starch. Our research demonstrates an effective preparation method for improving the quality of buckwheat-resistant starch and found that buckwheat-resistant starch has the role of adjusting the distribution of the intestinal flora and maintaining the health of the body.

## 1. Introduction

Buckwheat, a dicotyledonous plant, is a species within the genus *Fagopyrum*. Because of its cold, drought, and barren resistance, buckwheat is considered an important global grain crop [1]. The content of starch in buckwheat is approximately 67.8–80.7%. The content of amylose in starch is 33–34%. In addition to starch, buckwheat also possesses some unique flavonoid compounds, and the content of mineral elements and vitamins is dramatically higher in buckwheat relative to other grain crops [2,3,4]. Moreover, buckwheat has excellent nutritional value, and many components, such as linoleic acid, vitamins, dietary fiber, and mineral elements, provide health benefits. For example, linoleic acid in buckwheat can reduce serum cholesterol and blood lipids, regulate blood pressure, and promote the excretion of cholic acid and fecal sterols [5]. Different types of vitamins in buckwheat, such as vitamins B1 and B2, can prevent inflammation and diseases, such as neuritis and beriberi [6,7]. Nicotinic acid and flavonoids have been shown to have promising effects on cardiovascular disease, hypertension, hyperlipidemia, and eyesight health by improving vascular permeability and reducing blood lipids [8]. Dietary fiber in buckwheat can prevent diseases ranging from atherosclerosis and high cholesterol and promote intestinal peristalsis [9].

Additionally, abundant mineral elements are beneficial to the healthy function of buckwheat. Magnesium can inhibit atherosclerosis, hypertension, and hyperlipidemia by promoting fibrinolysis and suppressing thrombin formation [10]. Magnesium can also prevent myocardial infarction by increasing myocardial blood flow and regulating cardiac activity [11]. Potassium helps maintain the balance between osmotic pressure and moisture inside the body and thus effectively eliminates fatigue and enhances physical strength [12]. Selenium can also combine with heavy metal ions, reducing their toxic effects on the body. Meanwhile, selenium can protect the cell membrane and hemoglobin to avoid oxidation, enhance the immune regulating force and improve anti-aging function [13]. In addition, buckwheat has the components of protease inhibitors, phytic acid, and anti-nutritional factors, which can affect physiological functions, including blood cholesterol and fat accumulation-decreasing effects [14]. Given that starch is a main component of buckwheat, studies of resistant starch in buckwheat are few. In recent years, the effects of resistant starch on human health have attracted wide attention. Therefore, prepared resistant starch using buckwheat as a raw material could greatly improve the comprehensive utilization of buckwheat.

Prebiotics containing functional oligosaccharides, polysaccharides, plant nature extractions, protein hydrolysate, and polyols refer to nondigestible food ingredients that have beneficial effects on human health [15]. Prebiotics can facilitate the growth of *Bifidobacterium* and *Lactobacillus* in the intestinal canal [16,17]. Furthermore, prebiotics can promote the production of short-chain fatty acids, which can regulate the dynamic balance among intestinal bacteria [18]. Furthermore, Xing found that prebiotics can enhance the immunity of mice [19]. Resistant starch is an important prebiotic that has drawn wide attention. Resistant starch and its degradation products cannot be well digested in the small intestine. Based on differences in its structural and digestive properties, resistant starch can be divided into five types: RS1, RS2, RS3, RS4, and RS5. RS1 corresponds to physically trapped starch; RS2 corresponds to resistant starch granules; RS3 consists of retrograded starch; R4 is chemically modified starch; and R5 consists of compounds formed by amylose and lipids [20]. Because of their excellent thermal stabilities, RS3 has been the most widely used in applications among all types of resistant starch [21]. The current processing of resistant starch is not sufficient to meet the market requirement. Therefore, screening and delineating an appropriate preparation method is important for improving the quality and quantity of resistant starch.

Currently, the most common approach for processing resistant starch is autoclaved debranched treatment as well as acid and enzyme hydrolysis treatment. Autoclaved debranched treatment gelatinizes starch molecules under high temperature and pressure, during which the released amylose can formulate a more solid crystalline structure [22]. After autoclaved debranched treatment, the content of potato starch improved more than twofold [23]. Acid treatment hydrolyzes starch and forms a short molecular chain, resulting in starch rearrangement and resistant starch generation [24]. Enzymes, such as pullulanase, are capable of hydrolyzing the α-1,6 glucosidic bond of starch; resistant starch is then finally formed by improving the content of amylose [25]. In our previous research, we combined the autoclaved debranched and enzyme treatment, which we called autoclaving enzymatic debranching treatment (AEDT) [26], and prepared resistant starch in buckwheat along with acid hydrolysis treatment (AHT) [27]. The content of resistant starch was significantly increased by AEDT and AHT. The starch granular morphologies also changed after AEDT and AHT and were documented through scanning electron microscopy.

Resistant starches have promising effects on intestinal health, while the difference in physicochemical characteristics among different resistant starch preparation technologies was seldom studied. Therefore, the aim of the present study was to analyze differences in the physicochemical characteristics of the starch, including the crystalline property, amylose content, and anti-digestibility after AEDT and AHT. We investigated the effect of resistant starch on intestinal physiological function, including intestinal tract movement and intestinal microbes. Our research suggests a processing method for improving the content of buckwheat-resistant starch and the application of buckwheat-resistant starch on intestinal health.

## 2. Methods and Materials

### 2.1. Buckwheat-Resistant Starch

Raw buckwheat-resistant starch with a content of 23.5% (RS), AHT-resistant starch with a content of 29% (RS1), and AEDT-resistant starch with contents of 31.7% (RS2), 35.6% (RS3), 39.7% (RS4) and 45.5% (RS5) were prepared following previously described standard methods and were used for the analysis of physicochemical characters.

### 2.2. X-ray Diffraction Analysis of the Crystalline Mold of Buckwheat-Resistant Starch

A stepping scanning method was used to analyze the X-ray diffraction molds of buckwheat-resistant starch samples. The start and termination angle at 2θ for scanning was 4° to 65.0°, respectively, with a scanning speed of 0.06°/s. The sampling time, divergence slit, scattering slit, tube current, and voltage for X-ray diffraction were 1 s, 1°, 1°, 20 mA, and 30 kV, respectively.

### 2.3. Measurement of Amylose Content in Buckwheat-Resistant Starch

Standard amylose with contents of 0, 10, 20, 25, 30, and 35% was used to construct a standard curve. One hundred mg of standard amyloses were put into 1 mL of absolute ethyl alcohol and 9 mL of NaOH. The mixture was then dispersed under boiling water for 10 min and adjusted to a final concentration of 1 mg/mL. Iodine was added into a standard amylose solution; the absorbance value was then measured under 720 nm using a spectrophotometer. The standard curve was generated by placing amylose content on the X-axis and the absorbance value on the Y-axis. The amylose content of RS1–RS5 was determined according to the standard curve.

### 2.4. Anti-Digestibility of Buckwheat Starch

The anti-digestibility of RS1–RS5 was analyzed by an in vitro digestion model. Four hundred mg of resistant starch was added into sulfate buffer (pH 6.9) to a constant volume of 20 mL. α-Amylase was then added to the mixture and transferred to a dialysis tube. The dialysis tube was placed in the sulfate buffer (pH 6.9) at 37 °C. Digestive products were sampled at 0.5, 1, 1.5, 2, 3, 4, and 5 h. The sugar content was then determined by the phenol-sulfuric acid method [28]. Samples were mixed with a phenol solution and sulfuric acid; then, the absorbance was measured under 490 nm. The experiments were repeated three times.

### 2.5. The Influence of Buckwheat Resistance Starch on Mice Defecation

In total, 70 healthy mice with a weight of 20–25 g were randomly divided into 7 groups: buckwheat starch group (BS); low dosage of buckwheat resistance starch group (0.03 g/(10 g·bw), LBS); middle dosage of buckwheat resistance starch group (0.06 g/(10 g·bw), MBS); high dosage of buckwheat resistance starch group (0.12 g/(10 g·bw), HBS); negative group (NG), positive group (0.7 mg/(10 g·bw) of defecation capsule, PG) and constipation group (0.05 mg/(10 g·bw), CG) of compound diphenoxylate tablets). All the treatment groups were gavaged once each day, and after 15 d, experiment mice from all the treatments were fasting for 16 h and then gavaged with carbon ink. The time the first black feces appeared during each treatment was recorded, and the number of black feces within 5 h of each treatment was also recorded.

### 2.6. The Influence of Buckwheat Resistance Starch on Mice Intestinal Movement

Six treatments containing BS, LBS, MBS, NG, and CG were used to analyze the effect of buckwheat resistance starch on mice defecation. All the treatments were gavaged for 15 d, and then all the mice were fasting for 16 h. All the treatments were then gavaged with (0.05 mg/(10 g·bw) compound diphenoxylate tablets. After 0.5 h gavaged with carbon ink, the intestinal canal of each treatment was isolated, and the intestinal length was measured. The ink intestinal propulsion rate was regarded as the influence of buckwheat resistance starch on mice defecation. The ink intestinal propulsion rate (%) = ink intestinal propulsion length/intestinal total length × 100 [29].

### 2.7. The Influence of Buckwheat Resistance Starch on Mice Gut Microbes

In total, 125 mice were divided into five treatments containing BS, LBS, MBS, and NG to analyze buckwheat-resistant starch’s effect on mice gut microbes. All the treatments were gavaged for 14 d, 28 d, and 35 d, respectively. Microbes were isolated from the feces, colon, and caecum contents of mice from each treatment. All the isolated microbes were identified with 16S rRNA amplification primers.

## 3. Results

### 3.1. X-ray Diffraction

**Figure 1:**
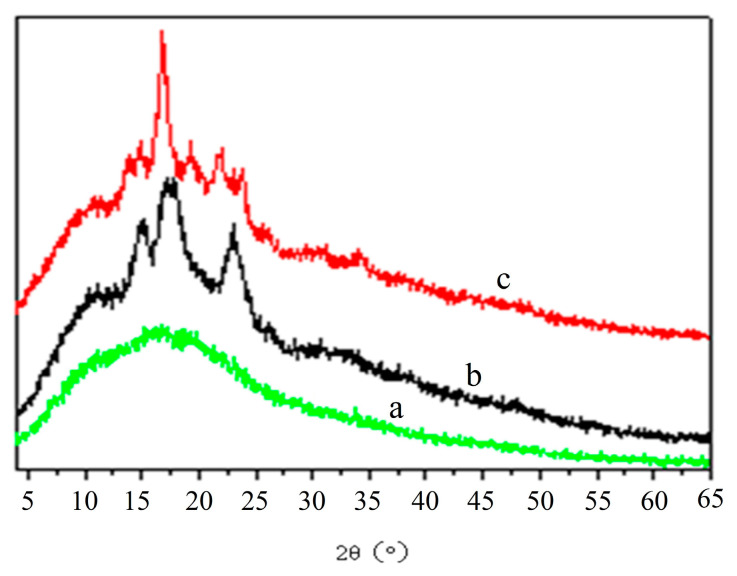
X-ray diffraction molds of raw and treated starches. a indicates buckwheat starch; b indicates resistant starch prepared using the acid hydrolysis method; c represents resistant starch prepared using the autoclaving enzymatic debranching method.

Buckwheat raw starch has obvious diffraction peaks with the diffraction angles at 15.10°, 17.14°, 17.92°, and 22.96°, which is characteristic of typical A-type crystals. AHT and AEDT significantly altered the X-diffraction molds of resistant starch. AHT resulted in strong diffraction peaks at 5.56°, 9.4°, 14.26°, 16.84°, and 19.86°, in which 5.56° is a typical type B crystal diffraction peak, and 14.26° and 19.86°crystal are typical type V crystal diffraction peaks. AEDT had highly similar diffraction peaks compared with AHT. AHT also exhibited a B + V type crystal structure, with the diffraction peaks at diffraction angles of 5.50°, 15.22°, 15.58°, 16.24°, 18.76°, and 19.84° (Figure 1).

### 3.2. Amylose Content in Buckwheat-Resistant Starch

The amylose content in AHT (RS1) and AEDT (RS2–5) was significantly higher than that in raw buckwheat (Table 1). Compared with AHT, the amylose content was dramatically improved after autoclaving enzymatic debranching treatment, which suggests that autoclaving enzymatic debranching was more suitable than AHT.

### 3.3. Anti-Digestibility Properties of Buckwheat Starch

**Figure 2:**
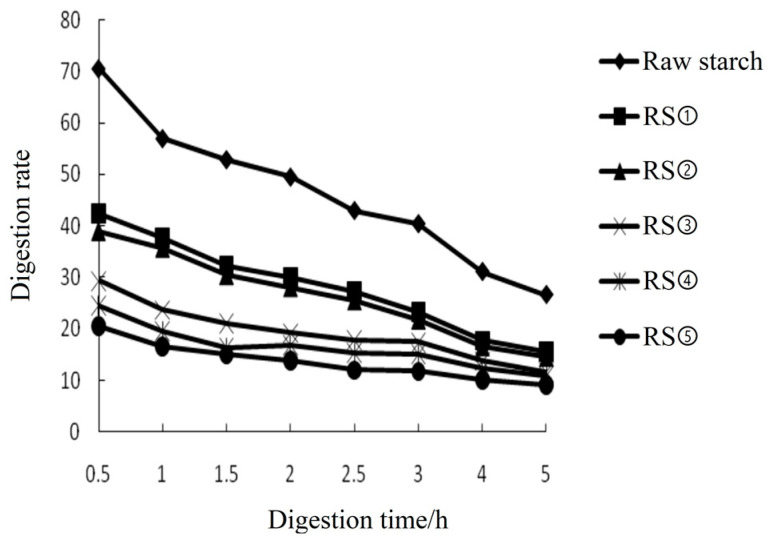
In vitro digestion rates of raw and resistant starches. The X-axis indicates digestion time; the Y-axis indicates digestion rates. RS① indicates preparation by the acid hydrolysis method with a resistant starch content of 29%; RS②–⑤ indicates preparation by the autoclaving enzymatic debranching method with the resistant starch contents of 31.7%, 35.6%, 39.7%, and 45.5%, respectively.

The amount of starch digestion products was positively related to digestion time among all of the buckwheat-resistant starch (Table 2). However, the starch digestion rate and yield were negatively related to the content of resistant starch (Figure 2). The higher content of resistant starch had a slower digestion rate and a lower number of products. Among buckwheat-resistant starch, RS5 had the highest resistant starch content but had the lowest digestion rate and the number of products. Thus, the results suggested that the higher content of resistant starch had stronger anti-digestibility to α-amylase and that AEDT provides an appropriate method for preparing buckwheat-resistant starch.

### 3.4. The Effect of Buckwheat Resistance Starch on Mice Defecation

Compared with other groups, the time the first black feces appeared in the CG group was longer than in other treatments. The time the first black feces appeared in HBS, MBS, and LBS was shorter than in the BS group, which indicated that gavaged with high, middle, and low dosages of buckwheat-resistant starch could promote mice defecation.

### 3.5. The Effect of Buckwheat Resistance Starch on Mice Intestinal Movement

Compared with the BS treatment, the ink intestinal propulsion rate was higher in three buckwheat resistance starch treatment groups, especially in the high dosage of buckwheat resistance starch group, which indicated that buckwheat resistance starch could promote bowel intestinal tract movement.

### 3.6. The Effect of Buckwheat Resistance Starch on Mice Gut Microbes

In total, four types of bacteria, including *Citrobacter*, *Enterococcus*, *Bifidobacterium,* and *Lactobacillus*, were isolated, identified, and selected for analysis.

**Figure 3:**
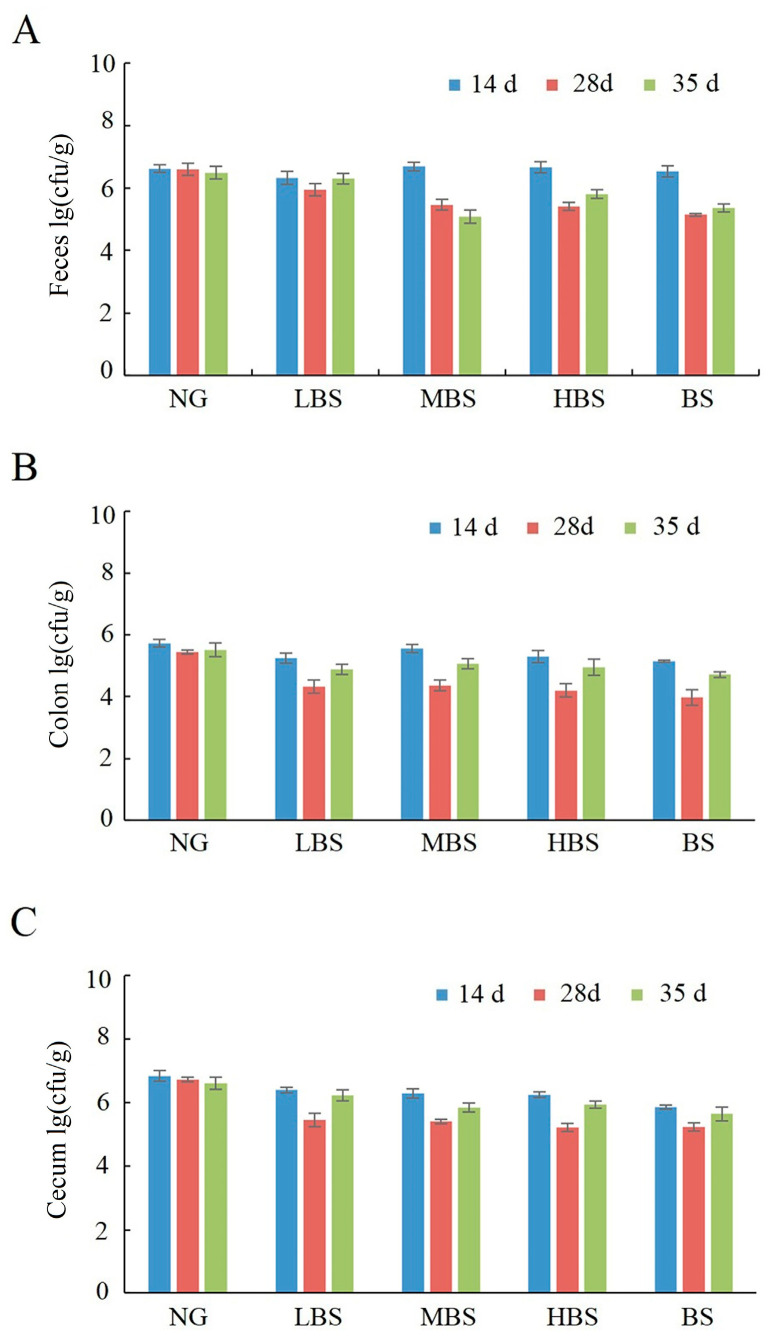
The effects of buckwheat resistance starch on the quantity of *Citrobacter* on feces (**A**), colon (**B**), and caecum (**C**). NG: negative control group; BS: buckwheat starch group; LBS: low dosage of buckwheat resistance starch group; MBS: middle dosage of buckwheat resistance starch group; HBS: high dosage of buckwheat resistance starch group.

The quantity of *Citrobacter* in the LBS, MBS, HBS, and BS groups was reduced in the colon and cecum tissues, as well as feces at 28 and 35 days compared with negative control (without gavaged of any types of starch). Compared with gavaged buckwheat starch, being gavaged with different dosages of buckwheat resistance starch could increase the quantity of *Citrobacter* in the colon and cecum tissues at 14, 28, and 35 days (Figure 3).

**Figure 4:**
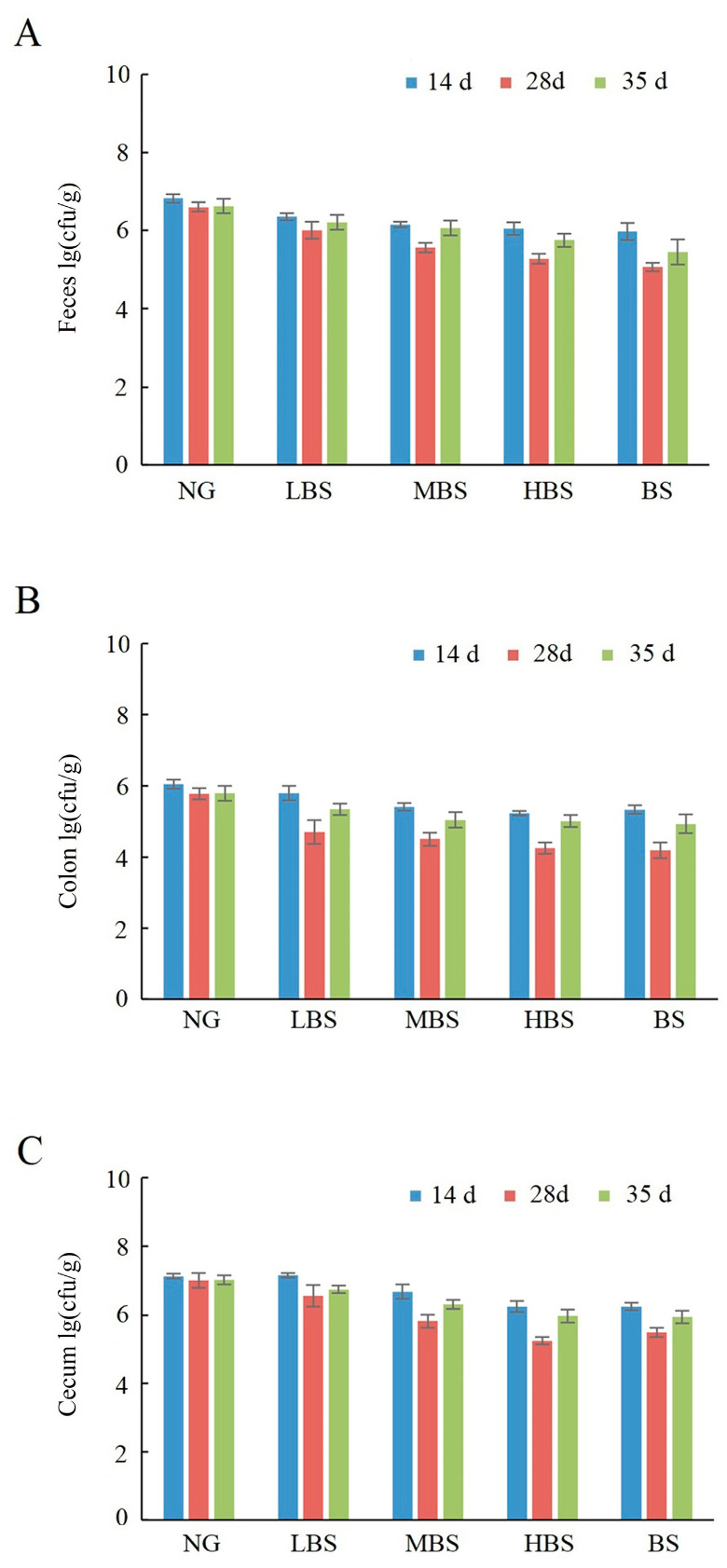
The effects of buckwheat resistance starch on the quantity of *Enterococcus* on feces (**A**), colon (**B**), and caecum (**C**). NG: negative control group; BS: buckwheat starch group; LBS: low dosage of buckwheat resistance starch group; MBS: middle dosage of buckwheat resistance starch group; HBS: high dosage of buckwheat resistance starch group.

A similar phenomenon had been found in *Enterococcus*. The quantity of *Enterococcus* was reduced after being gavaged with buckwheat starch and different dosages of buckwheat resistance starch at all time points. The quantity of *Enterococcus* was lower in the three buckwheat resistance starch groups than in the buckwheat starch group (Figure 4), which means that the resistance starch had an inhibition effect on *Enterococcus*.

**Figure 5:**
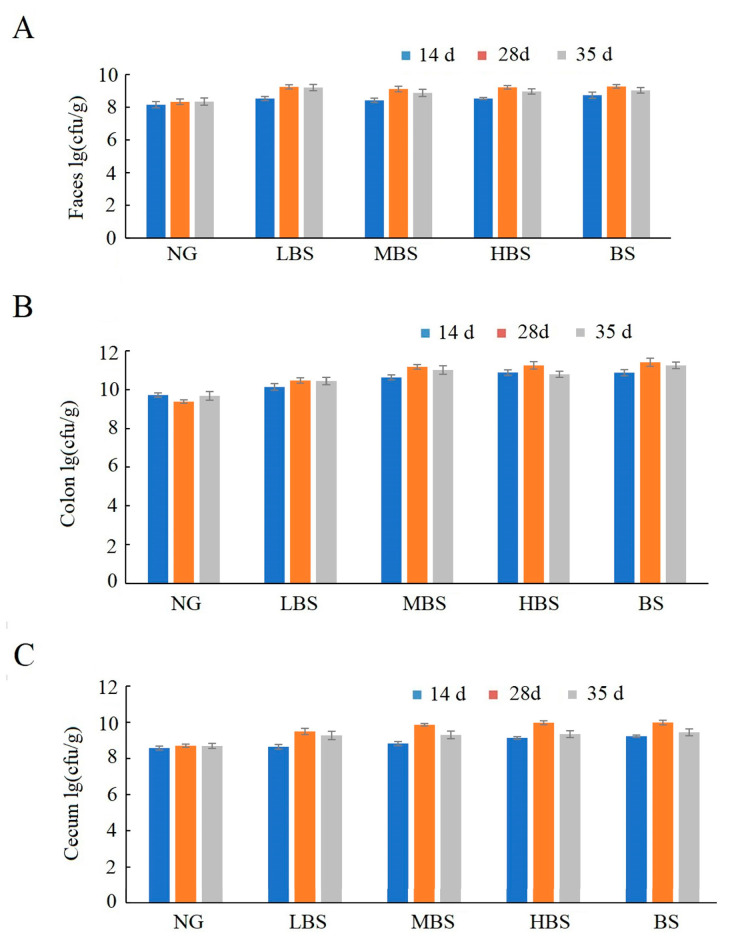
The effects of buckwheat resistance starch on the quantity of *Bifidobacterium* on feces (**A**), colon (**B**), and caecum (**C**). NG: negative control group; BS: buckwheat starch group; LBS: low dosage of buckwheat resistance starch group; MBS: middle dosage of buckwheat resistance starch group; HBS: high dosage of buckwheat resistance starch group.

**Figure 6:**
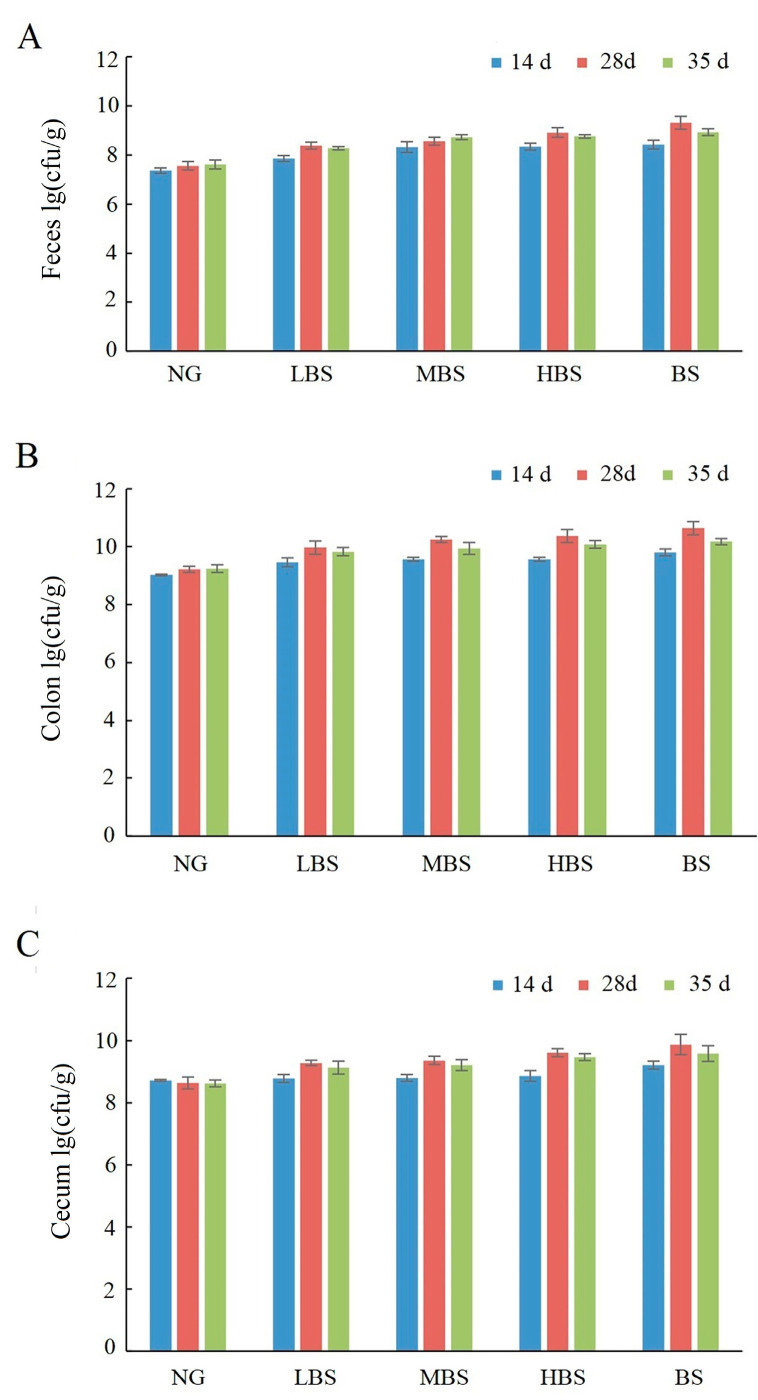
The effects of buckwheat resistance starch on the quantity of *Lactobacillus* on feces (**A**), colon (**B**), and caecum (**C**). NG: negative control group; BS: buckwheat starch group; LBS: low dosage of buckwheat resistance starch group; MBS: middle dosage of buckwheat resistance starch group; HBS: high dosage of buckwheat resistance starch group.

For *Bifidobacterium* and *Lactobacillus*, the quantity alteration was different with *Enterococcus* and *Citrobacter*. The quantity of *Bifidobacterium* and *Lactobacillus* in the buckwheat starch group and buckwheat resistance starch groups was higher than without gavaged of any types of starch. Compared with the buckwheat starch group, being gavaged with different dosages of buckwheat resistance starch could increase the quantity of *Bifidobacterium* and *Lactobacillus* (Figure 5 and Figure 6).

## 4. Discussion

Resistant starch appears to play an important role in food applications and physiological functions. On the one hand, it is used to develop a variety of functional-resistant starch products in the food and pharmaceutical fields. The addition of resistant starch to flour can markedly improve the quality of noodles, including increasing their brightness and decreasing their digestibility and the flavor of bread [30,31]. On the other hand, resistant starch could also improve the puffed properties of food by reducing the high hardness and low brittleness caused by the puffing process. Furthermore, adding resistant starch to fried food could reduce the oil content and improve the nutritional value, color, hardness, brittleness, and the content of dietary fiber [32,33]. Additionally, resistant starch could be used as thickeners to improve the sensory properties and health function of beverages [34]. After the addition of resistant starch, the content of fat decreased in the yogurt, and the number of probiotics was altered; in addition, the nutritional value of yogurt was improved [35]. In the medical field, resistant starch can be used as vectors to embed drugs and probiotics. Microencapsulation can delay the release of drug ingredients or probiotics in the body. Using resistant starch as microcapsule wall material could improve the efficacy of drugs or probiotics [36,37,38].

Moreover, previous studies suggest that resistant starch may play important roles in physiological function and the management of body sugar and lipid metabolism. Resistant starch can regulate glucose and insulin levels, improving the balance between glucose and lipids in the body. The intake of food containing resistant starch could help control diabetes by reducing blood glucose and increasing insulin sensitivity and intestinal hormones [39]. Hence, the use of resistant starch can help prevent and treat diabetes [40]. In addition, resistant starch can reduce the content of triglycerides and cholesterol in the body by increasing the excretion of steroids and excreta. It can affect the size of fat cells in high-fat diet mice [41]. For example, Cheng found that resistant starch could dramatically decrease the concentration of triglycerides and total cholesterol in high-cholesterol mice [42]. In other words, resistant starch can manage body weight by reducing the energy density of food, promoting the decomposition of adipose tissue, and increasing satiety. Muffins composed of a high content of resistant starch can stimulate body satiety and extend digestion time, promoting weight loss. The weight of mice was significantly decreased after feeding food containing resistant starch [43]. Resistant starch can reduce the incidence of gallstones through the regulation of insulin secretion [44].

As a prebiotic, resistant starch is vital to the regulation of gut microbes and intestinal health. Resistant starch can promote the growth and reproduction of beneficial intestinal microbes, such as *Bifidobacterium* and *Lactobacillus*, but inhibit the growth of acidophilic bacteria, such as *Escherichia coli*, *Clostridium perfringens* and *Bacteroides*, which might stem from the structure of the resistant starch surface [45,46]. Barczynska found that corn-resistant starch could increase the number of bacteroidetes and actinomycetes but decrease firmicutes in the feces of obese children [47]. Resistant starch could also improve intestinal function and prevent the incidence of intestinal diseases, including ulcerative colitis, diarrhea, rheumatoid arthritis, and gastrointestinal disorders [48]. Lastly, resistant starch can reduce the expression of inflammatory cytokines, ileum, and colon inflammation lesions in sick mice.

Understanding the mechanism by which resistant starch promotes health is important for its future applications. The addition of resistant starch into an oral rehydration solution could significantly reduce fecal fluid losses and the duration of diarrhea with cholera [49]. It could also prevent the digestion of amylase in the small intestine as well as the rapid fermentation of short-chain fatty acids (SCFAs) by luminal bacteria in the colon [50]. SCFAs could then improve the environment of the intestinal tract, facilitate the growth and reproduction of beneficial microbes, and further inhibit or even kill harmful microbes [51]. SCFAs helped improve the absorption of water and sodium, provide an additional energy source, decrease fecal stool losses, and enhance mucosal function and recovery [52]. The absorption of sodium by SCFAs primarily depends on Na-H exchange [53]. Moreover, in the colon, SCFAs can inhibit the secretion of chloride, which is crucial for fluid secretion in cholera and other types of secretory diarrhea. The most effective SCFA is butyrate. The production of butyrate is positively associated with the content of resistant starch in taro [54]. Butyrate can facilitate the absorption of sodium and water in the colon [55]. In addition, butyrate can inhibit intestinal malignant cell transformation and prevent colorectal cancer. Butyrate also had excellent inhibiting effects on the proliferation of colorectal cancer cells at the G1 stage [56].

The formation of resistant starch was influenced by amylose; therefore, using buckwheat starch to prepare it could reduce the waste of resources and improve the utility and value of buckwheat. Hence, developing an effective method for improving the content of resistant starch is important. Previously, we used AHT and AEDT to prepare resistant starch; this study primarily analyzed the physicochemical characteristics. Finally, we confirmed that AEDT results in the highest resistant starch content and the most stable structure.

Different preparation methods can affect the crystalline structure of resistant starch. In this study, the crystalline structure of raw buckwheat starch changed from A to B + V after AHT and autoclaving enzymatic debranching treatment. Similar observations have been made in waxy maize. For example, Shi found after pullulanase debranching that the crystalline structure mold of raw starch changed from A to B + V [57]. Figure 1 shows that the relative crystallinity in raw starch was significantly lower than that in AHT and AEDT. This finding might stem from the rearrangement of short-chain starch molecules derived from starch debranching.

We also found that the content of resistant starch was positively related to amylose. This pattern might stem from the fact that the amylose molecules form a double helix structure based on intermolecular forces, which result in the generation of resistant starch. Guraya found that increasing amylose facilitated the linkage of amylose molecules and promoted the formation of resistant starch [58]. However, the length of the starch chain might also influence the formation of resistant starch. Although more amylose could be generated through excessive debranching, the content of resistant starch did not increase. Eerlingen also found suitable lengths of starch chains were necessary for crystalline formation [59]. In this study, we found that the digestion rate and products were negatively related to the content of resistant starch. Gonzalez-Soto found that the digestion rate of α-amylase in banana-resistant starch prepared by autoclaved debranched was slower than that in raw banana starch [60]. The anti-digestibility of resistant starch primarily depended on its ordered arrangement and crystalline structure. Shin reported that resistant starch has amorphous regions and an imperfect crystal structure, which appeared to inhibit the digestive function of α-amylase, thereby reducing the digestion rate [61].

Compared with buckwheat starch treatment, gavaged with buckwheat resistance starch could promote bowel intestinal tract movement, which might be due to the buckwheat resistance starch could ferment into short-chain fatty acids in the colon. These short-chain fatty acids could stimulate the intestinal wall, promote the intestinal absorption of water and cause intestinal movement, thereby shortening the retention time of the feces in the gut.

Future work could focus on deeply investigating the application of the selected buckwheat-resistant starch on human health, such as regulating the metabolism of blood lipids, blood sugar, or cholesterol. Moreover, developing buckwheat-resistant starch products was also a concern.

## 5. Conclusions

The physicochemical properties of buckwheat-resistant starch were altered after AHT and AEDT. The crystalline mold in buckwheat raw starch changed from A to B + V after AHT and AEDT. As the resistant starch content increased, the amylose content increased. In vitro digestion simulation experiments showed that the digestion rate and products were negatively related to the content of resistant starch. The buckwheat-resistant starch can promote bowel intestinal tract movement. The quantity of intestinal microbe was regulated by buckwheat-resistant starch. Our research suggests a novel approach for preparing resistant starch with high content and applying buckwheat-resistant starch to intestinal health.

## Figures and Tables

**Table 1:** Amylose content of raw and treated starches.

Sample	Amylose Content (%)
Raw buckwheat	31.428 ± 0.2
RS①	43.616 ± 0.6
RS②	56.100 ± 0.8
RS③	58.204 ± 0.6
RS④	59.505 ± 0.9
RS⑤	59.109 ± 1.1

RS① indicates preparation using the acid hydrolysis method with a resistant starch content of 29%; RS②–⑤ refers to preparation using the autoclaving enzymatic debranching method with resistant starch contents of 31.7%, 35.6%, 39.7%, and 45.5%, respectively.

**Table 2:** Digestion products of raw and treated starches.

Sample	Digestion Products in Different Times of Samples/mg							
	0.5 h	1 h	1.5 h	2 h	2.5 h	3 h	4 h	5 h
Raw buckwheat	35.28	56.96	79.28	98.92	107.40	121.20	124.41	133.10
RS①	21.17	37.63	48.34	59.79	68.09	69.74	70.74	77.54
RS②	19.48	35.73	45.59	56.12	63.75	65.27	66.19	72.45
RS③	14.69	23.69	31.63	38.56	44.63	52.23	55.12	58.03
RS④	12.32	19.57	24.69	33.84	38.42	45.59	49.26	54.60
RS⑤	10.34	16.60	22.54	27.68	30.08	35.34	40.82	45.51

RS① indicates preparation by the acid hydrolysis method with a resistant starch content of 29%; RS②–⑤ refers to preparation by the autoclaving enzymatic debranching method with resistant starch contents of 31.7%, 35.6%, 39.7%, and 45.5%, respectively.

## Data Availability

Data is contained within the article.
